# Oscillating Electric Field Measures the Rotation Rate in a Native Rotary Enzyme

**DOI:** 10.1038/srep45309

**Published:** 2017-03-27

**Authors:** Csilla-Maria Ferencz, Pál Petrovszki, András Dér, Krisztina Sebők-Nagy, Zoltán Kóta, Tibor Páli

**Affiliations:** 1 Institute of Biophysics, Biological Research Centre, Hungarian Academy of Sciences, Temesvári krt. 62, 6726, Szeged, Hungary

## Abstract

Rotary enzymes are complex, highly challenging biomolecular machines whose biochemical working mechanism involves intersubunit rotation. The true intrinsic rate of rotation of any rotary enzyme is not known in a native, unmodified state. Here we use the effect of an oscillating electric (AC) field on the biochemical activity of a rotary enzyme, the vacuolar proton-ATPase (V-ATPase), to directly measure its mean rate of rotation in its native membrane environment, without any genetic, chemical or mechanical modification of the enzyme, for the first time. The results suggest that a transmembrane AC field is able to synchronise the steps of ion-pumping in individual enzymes via a hold-and-release mechanism, which opens up the possibility of biotechnological exploitation. Our approach is likely to work for other transmembrane ion-transporting assemblies, not only rotary enzymes, to determine intrinsic *in situ* rates of ion pumping.

There is a class of membrane-attached macromolecular assembly, which plays a crucial role in living processes and disease, whose action involves true, full-cycle rotation of certain parts, the rotor, relative to other parts, the stator^1^,^2^,^3^,^4^,^5^,^6^. Direct measurement of rotation in such rotary enzymes was so far impossible without genetic, chemical and even physical modification. In all such studies the rotary enzyme had to be removed from its native membrane environment, and in most cases some subunits were also removed^5^,^7^,^8^,^9^,^10^,^11^,^12^,^13^. Even the (so far) least invasive and least perturbing single molecule fluorescence resonance energy transfer approach to detect the rotation required the chemical modification of the rotary enzyme, namely binding of fluorescent donor and acceptor groups and reconstitution of the enzyme into artificial liposomes as “quasi-native environment”^14^. In the present study we use spherical single-layer membrane vesicles, with a mean diameter of 300 nm, formed from yeast vacuoles as described earlier^15^. These vesicles contain high concentration of properly oriented vacuolar proton-pumping adenosine-triphosphate (ATP) hydrolase (vacuolar proton-ATPase, V-ATPase), on which we measure ATPase activity^15^. In addition, since ATP is added to pre-formed stable vacuolar vesicles, reversely (inside-out) oriented V-ATPases, if any, remain inactive and do not contribute to the measured ATPase activity. An AC-field is applied to the vesicle suspension in a rectangular cuvette with platinum electrodes. For the first time: (*i*) we apply an AC field to V-ATPase; (*ii*) we reveal a narrow, resonance-like frequency response to the AC field that has never before been seen for any enzyme, and cannot be interpreted within the framework of electro-conformational coupling theory^16^,^17^,^18^; and (*iii*) the resonance-like effect of the AC field on transmembrane proton movement allows us to determine the intrinsic mean rotation rate in native V-ATPase directly: it is 13.2 ± 0.5 Hz (under well defined conditions).

As a family, the normal function of V-ATPase is to pump protons across specific biomembranes. It is a key rotary enzyme in all eukaryotic cells, acidifying intracellular compartments and the extracellular space in some tissues^1^,^3^,^4^,^6^. A class of proteins including the proteolipid *c*-ring subcomplex has functions independent from V-ATPase activity^19^,^20^,^21^,^22^. The V-ATPase is also a potential therapeutic target for several diseases^1^,^4^,^23^,^24^. Functionally, the V-ATPase works in the opposite sense to the better known F-ATP synthase^2^,^3^,^5^,^7^,^10^,^12^,^13^,^25^,^26^. Proton transport is energised by the chemical energy of ATP, which is transduced into mechanical force to rotate a special group of subunits relative to the rest of the protein complex. Both F- and V-ATPases are true molecular engines^3^,^10^,^25^,^26^,^27^. A complementary arrangement of proton-binding sites in the V-ATPase, namely glutamic acid (Glu) on the rotor – one Glu on each *c*-ring subunit – and proton-conducting hemichannels at the interface with a stator subunit, ensures active proton transport across the lipid bilayer driven by protein rotation^4^,^6^,^27^ (Fig. 1, Supplementary Videos 1 and 2). As in the F-ATPase, ATP hydrolysis and proton transport are strongly coupled via the rotary mechanism: there is no active proton transport without ATP hydrolysis and blocking protonation-deprotonation of the unique Glu residue stops ATP hydrolysis^1^,^5^,^6^,^10^,^12^,^23^,^24^.

## Results and Discussion

In typical studies of rotation in membrane-attached rotary enzymes up until now, one of the subunits, e.g., of the stator, was covalently bound to a solid support, whereas a subunit of say the rotor was covalently labelled with a gold bead or a fluorophore in order to visualise the rotation with a microscope. Because these modifications, and even fluorescent labelling and reconstitution, potentially added or removed free energy barriers to rotation, results varied greatly depending on the type of modification and other conditions^3^,^5^,^7^,^8^,^9^,^10^,^11^,^12^,^13^,^14^. The inherent limitations of these current approaches prompted us to explore a very different route. Our idea was straightforward: because the periodicity of vectorial charge movement relates directly to that of rotation, an oscillating transmembrane potential with frequency matching the turnover rate of charge movement, should have maximum effect on enzyme activity. The challenge was to optimise the conditions to obtain significant effects. Measurements were made on membrane vesicles formed from yeast vacuoles. Therefore, the V-ATPase remained in its native membrane environment. ATP was hydrolysed only by properly oriented (right-side-out) V-ATPase in the vesicles. The specific and highly potent inhibitor concanamycin A (ConcA) was used to determine the V-ATPase contribution to total ATPase activity^15^. ConcA binds to the intramembranous domain of the V-ATPase, blocking rotation and hence proton transport and ATP hydrolysis^15^,^23^,^24^,^28^. Although V-ATPase sits in its native membrane environment, a static membrane potential is not present in our vesicles, as opposed to vacuoles in living yeast cells. Two platinum electrodes were immersed in the vesicle suspension, in a flat cuvette. In such a system a transmembrane potential is established across the vesicle membrane because of the insulating property of the lipid bilayer.

**Figure 2:**
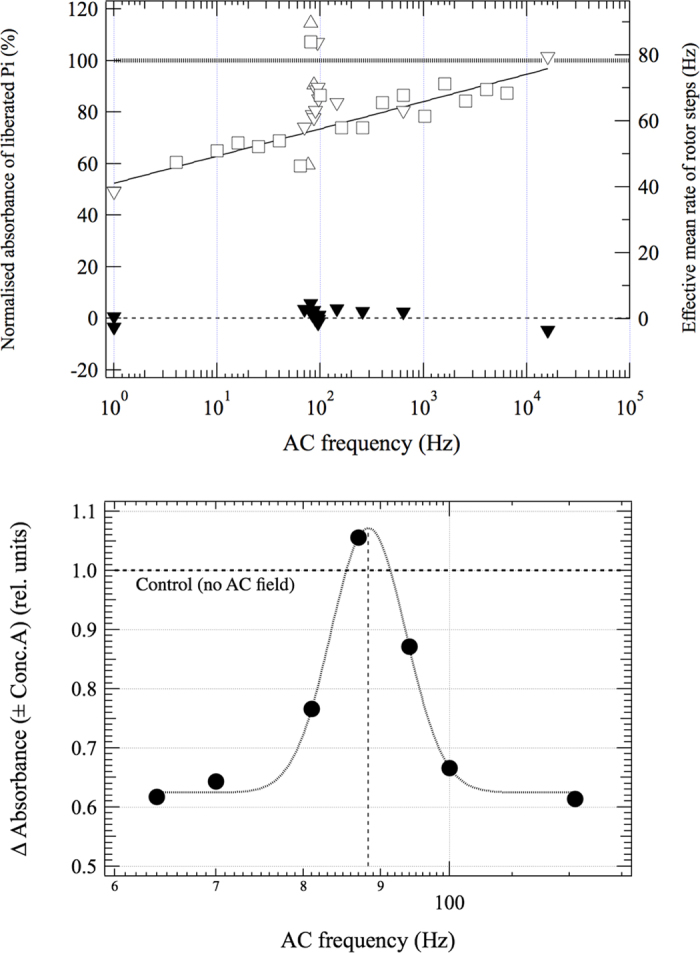
V-ATPase in an oscillating electric field. Frequency dependence of the effect of oscillating electric field on ATP hydrolysing activity of yeast vacuolar vesicles. Conditions: reaction time, 10 min; temperature, 20 °C; substrate in excess. Top: Frequency dependence for in the presence (solid symbols) and absence (open symbols) of the specific V-ATPase inhibitor concanamycin A. Different symbols mean different set of experiments. The effective mean rate of rotor steps (right axis) is defined in the text. Bottom: Delta absorbance (±inhibitor) over a narrow frequency range around the sharp peak (single set of experiments). The line fit in the top panel excludes the sharp peak whereas the Gaussian fit in bottom panel is restricted to the peak. (Statistics on the fitting parameters are given in the text).

**Figure 3:**
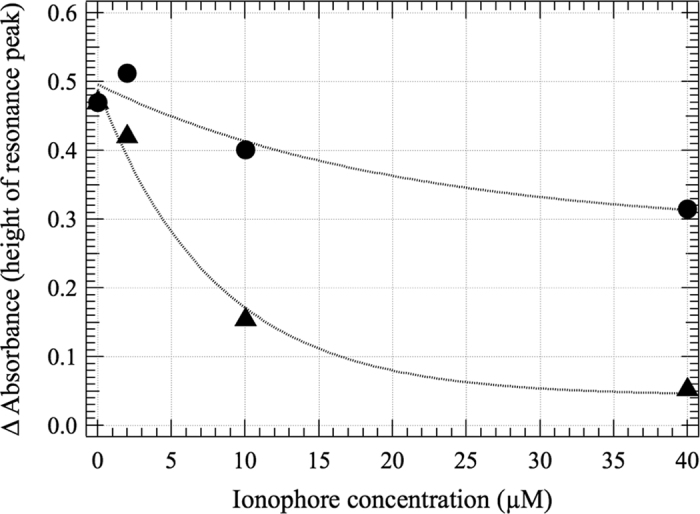
Short circuiting the transmembrane potential. Amplitude of the sharp peak (Fig. 2, bottom) as a function of ionophore concentration (conditions described under Fig. 2). The amplitude is measured relative to the broad background, which increased with increasing ionophore concentration. The ionophores were FCCP (carbonylcyanide p-trifluoromethoxyphenylhydrazone, triangles) and GrA (gramicidin A, circles).

ConcA removed the dependence of ATPase activity on AC field, proving that it is the V-ATPase that responds to the AC field (Fig. 2, Top). It should be noted that the high ConcA sensitive fraction of the ATPase activity proves that most of the V-ATPases are properly oriented in these vesicles^15^. We discovered a narrow peak in V-ATPase activity as a function of frequency of the AC field (Fig. 2). Such a resonance like response was not expected at all, but this is what we got. The peak is located between 81–88 Hz in five independent data sets. The position, amplitude and half-width at half-height (HWHH) of the narrow peak were 85.2 ± 3.5 Hz (*n* = 5), 41 ± 20% (*n* = 5) and 4.6 ± 1.4 Hz (*n* = 4) (errors are standard deviations), respectively, when fitting with a Gaussian function. Fitting instead with a Lorentzian function gave 86.0 ± 2.9 Hz (*n* = 5), 42 ± 17% (*n* = 5), 3.0 ± 2.0 Hz (*n* = 4), respectively. We observed a linear dependence, in a linear-log plot, over the frequency range 1 Hz to 20 kHz, excluding the narrow peak, with lower frequencies being the more inhibitory and frequencies above 10 kHz having no effect (Fig. 2, Top). The effect of short circuiting the transmembrane potential with pore-forming ionophores was measured in the 76–100 Hz region. It reversed the effect of the AC field: with increasing ionophore concentration the activity values approached the control (no AC) situation. (Fig. 3). (Note that in the absence of an AC field, enhancement of V-ATPase activity is expected because pores would dissipate the proton gradient). These observations prove that the AC field must act on V-ATPase-related charge movements in the direction of the membrane normal. In all previous experiments on frequency dependence of the effect of an AC field on activity of other enzymes very broad peaks were observed, even orders of magnitude wide, and the curves were fitted according to electro-conformational coupling theory^17^,^18^,^29^. However, the ratio of width to position of the narrow peak in Fig. 2 falls outside the region of applicability of that theory. The reason is most certainly that proton transport is driven by ATP hydrolysis, even at the resonance peak, and not by the AC field. Indeed, as opposed to other studies^17^,^18^,^29^,^30^ the AC field in our system inhibits rather than stimulates ATP hydrolysis, except for the resonance peak.

**Figure 4:**
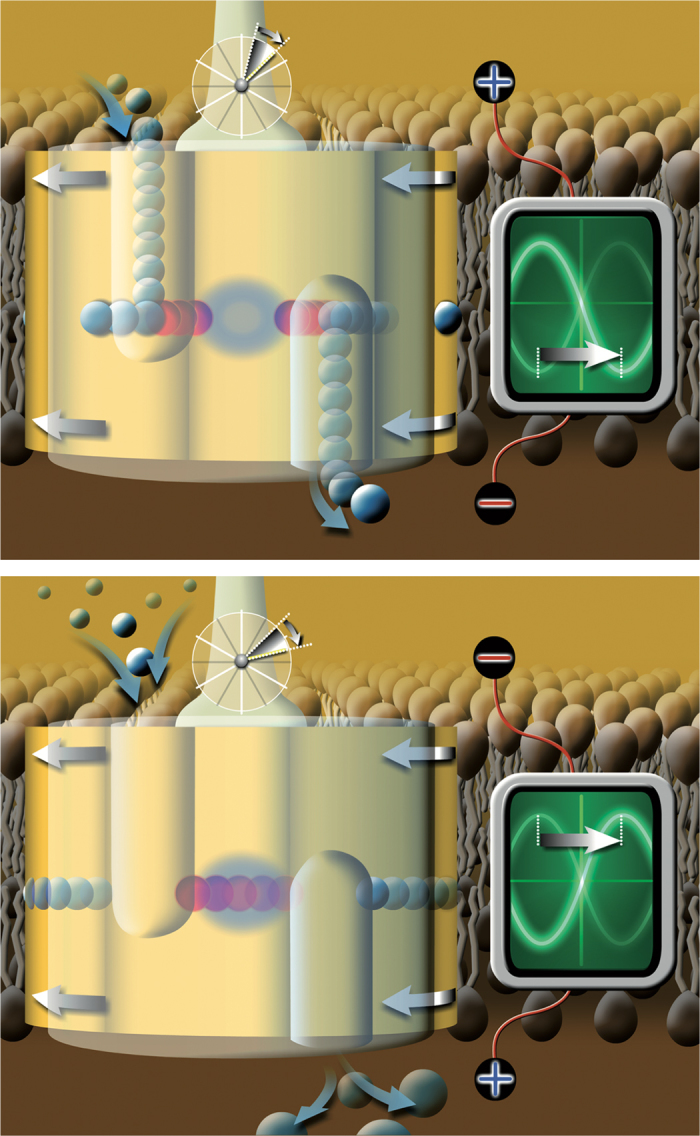
Matching of the steps of proton-pumping to the phases of the oscillating electric field. Effect of oscillating (AC) transmembrane electric field on V-ATPase activity, when the AC frequency matches the rate of the 60° rotor steps. Top: Protonation-deprotonation of the rotor Glu residues is possible, because protons occupy the hemichannels. If the AC field is in the “right” phase it favours, otherwise it opposes, proton transport. Bottom: Protonation-deprotonation of Glu residues is not possible because the hydrophilic hemichannels are empty of protons. In this rotor phase the AC field has no effect on transport, even in the “wrong” phase.

It would need real-time frequency scans to be able to fit a theoretical model to the whole frequency dependence. Therefore, we do not attempt a complete theoretical description of the data. In particular, we do not offer an explanation for the linear dependence. However, there are very strong arguments for the effect being a consequence of the transmembrane AC field acting by periodically changing the effective pK_a_ values of the rotor Glu residues, and hence their protonation-deprotonation rates. The largest charge movements directed along the membrane normal in the V-ATPase are those of the pumped protons, which are connected with protonation-deprotonation of the single Glu residues on each of the 6 rotor subunits. But how is it that the bidirectional AC field does not always inhibit unidirectional proton transport? The rotor Glu residues are protonated, i.e. uncharged, when in contact with the lipid chains, and their protonation state only can change at the interface between the rotor subunit *c*-ring and the stator subunit *a*. Close inspection of the hemichannels at this interface^25^,^27^ shows that in each rotation step (equivalent to rotation by one *c*-ring subunit) there is a phase in which the protonation state of any Glu residue can change (“active” phase), and in another phase cannot change (“dead” phase) (Fig. 4). If the two phases have comparable temporal duration, an AC field does not inhibit the enzyme if its frequency matches that of the periodicity of the protonation (Supplementary Video 3). This is because if the “wrong” phase of the AC field (pointing in the direction opposite to proton transport) matches the “dead” phase of the rotation there is no inhibition, consequently the “right” phase of the sine matches the “active” phase and therefore also there is no inhibition, it might even enhance transport. This explanation also means that the AC field is strong enough and blocks rotation in the “wrong” phase of the sine wave. This hold-and-release effect of the AC field changes the stochastic stepping motion of the rotor^31^ into a more regular periodic motion. This is synchronised stepping rotation because all the V-ATPases in the same side of the vesicles (with respect to the electrodes) are released at the same time. Of course, the opposite phases of the sine are periodically “right” and “wrong” at the different poles of the membrane vesicles with respect to direction of the electrodes, meaning that the two “poles” of the vesicles have the same behaviour when averaged over a time longer than the periodicity of both the sine wave and the rotor steps. It follows that the AC field periodically blocks rotation, hence inhibits the enzymatic activity too, of any single enzyme, except in the high-frequency limit. The inhibition vanishes if the following conditions are both met: (*i*) the frequency of the AC field matches the effective mean rate of rotor steps and (*ii*) the duration of the non-inhibitory (“right”) and the inhibitory (“wrong”) phases of the AC field match that of the “active” and “dead” phases of the protonation-deprotonation process, respectively. The double – frequency and phase – matching condition (aided by a hold-and-release mechanism) is quickly lost when the AC frequency goes down or up from the resonance frequency, which explains why the peak is narrow. The Supplementary Videos give simple, mechanistic illustrations for the timing of binding ATP, releasing ADP and inorganic phosphate, protonation-deprotonation steps, and proton transport as a function of rotation under normal conditions, and under influence of a transmembrane AC field with frequency either matching, or being slower or faster than, the intrinsic rate of rotation of the V-ATPase with excess ATP and assuming *m* = 6 *c*-ring subunits.

It follows that the frequency of the narrow peak, 86.0 ± 2.9 Hz, is that of rotation of the rotor by one proton-carrying monomeric *c*-ring subunits, i.e. 360°/*m* in the presence of the AC field oscillating at the same frequency. It should be noted that at resonance the ATPase activity is higher in the presence than in the absence of the AC field. The overshoot is ~9% (when averaging it from the Lorentzian fits), which could be caused by electro-conformational coupling^17^,^18^,^29^ and by reducing energy barriers of the protonation-deprotonation process. So, the AC field can enhance enzymatic activity only by ~9% despite being strong enough to decrease it by up to ~50% outside the resonance region. This, again, means that the enzyme is driven by the catalytic steps (of ATP hydrolysis) and not by the AC field.

Since the ATPase activity at the matching AC frequency (86 Hz) is at 109% of the control level, the intrinsic mean rate of the 60° rotation steps of the rotor (shortly rotor steps) is 79.1 ± 2.7 Hz corresponding to 100% activity, i.e. to the control with no AC field. Making use of the strong coupling, hence the proportionality between the ATPase activity and the mean rotation rate, the relative ATPase activity can be used to calculate the mean effective rates of rotor steps (see the right y-axis of Fig. 2, Top). In most but not all studies it is measured or assumed that *m* = 6 in V-ATPase^1^,^3^,^6^,^15^,^22^,^23^,^32^. Using the Lorentzian fits and taking *m* = 6, the mean frequency of the full-cycle 360°-rotation is 13.2 ± 0.5 Hz (s.d., *n* = 5). It should be noted that this result is independent of the presence of any inactive (e.g. reversely oriented) V-ATPases. This mean rotation rate agrees very well with a recent indirect estimate of ~14 Hz on the same experimental system but using the inhibition curve of ATP hydrolysis of V-ATPase by ConcA^15^. The latter estimate was based on several assumptions about the binding of the inhibitor. In addition, the average width of the Lorentzian (i.e., 1/(1 + *ω*^2^/*k*^2^), where *ω* is the angular frequency^17^,^29^ yields a relaxation rate constant *k* = 19 ± 12 Hz (s.d., *n* = 5). This can be interpreted as the rate of protonation of any single Glu residue, which is again the frequency of full-cycle 360°-rotation. These agreements give us full confidence that we have indeed measured the intrinsic mean rotation rate in an intact and unmodified rotary enzyme in its native membrane environment, for the first time.

According to our model, except for the region of the narrow peak, the AC field inhibits the enzyme by interfering with proton transport and hence rotation, via the strong coupling of hydrolysis and transport. If the AC field is strong enough to block rotation during the “wrong” phase of the cycle, the theoretical reduction in activity in the low-frequency limit would be ~50%, because any single enzyme is exposed to the non-inhibitory “right” phase and the inhibitory “wrong” phase of the sine wave for the same length of time (i.e. half the reaction time). (Note that in the low-frequency limit ~50% of the V-ATPases are always blocked like in a static field). During the “wrong” phase of the AC field the enzyme is stopped, during the “right” phase it works at 100–109% of its intrinsic rate, resulting in an average of ~45–50% of reduction of ATPase activity and effective mean rate of rotor steps (Supplementary Video 4). If the frequency of the AC field is much higher than that of the relaxation rate of protonation-deprotonation, the AC field should cause no inhibition (Supplementary Video 5). These predictions for the low- and high-frequency limits agree with our data (Fig. 2). As concerns the shape of the broad frequency dependence of the relative activity and the effective mean rotation rate outside the resonance-like region (Fig. 2), if there would be no other factors than the relaxation rates the function should be monotonic, and it is. However, it would need a detailed theoretical model combined with real-time frequency scans of the spontaneous ATPase activity to quantitatively describe the whole frequency dependence, and we are working on this. Concerning future studies and biotechnological application, we note that a transmembrane AC field is capable of synchronising rotation in individual V-ATPase molecules, on a 360°/*m* quantisation grid, by blocking them in the “wrong” and releasing them in the “right” phase, which is a hold-and-release mechanism. The present method could also work on other transmembrane ion-transporting systems, not only rotary enzymes, to determine the intrinsic rate of ion pumping.

## Materials and Methods

### Experimental Design

The use of vacuolar membrane vesicles ensures that native V-ATPase remains hosted in its membrane environment. Since the periodicity of vectorial charge movement in V-ATPase relates directly to that of rotation, an oscillating transmembrane potential with frequency matching the mean turnover rate of charge movement, should have maximum effect on enzyme activity. The effect can be amplified when measuring ATPase activity over several minutes. However, concentrations (of the vesicles, substrate, inhibitor), buffer conditions, pH, temperature, reaction time, applied voltage and sample geometry all had to be optimised for effect. In addition, because of lack of knowledge on what kind of frequency response to expect and in which frequency region, the experiment was of a trial-and-error type at the beginning.

### Materials, Sample Preparation and Measurement

Native yeast vacuolar membrane vesicles were prepared as in reference^15^, which also describes assay conditions and activity measurements. Most water-soluble components in the vacuoles were removed by washing. All standard chemicals were of analytical grade purity. The sample cell was immersed in a thermostatted water bath and its temperature was monitored and kept constant at 20 °C during the reaction time. The reaction was stopped after 10 min and phosphate liberated from ATP was assayed photometrically. ATP was in excess even at the end of the reaction period, so that the rate of ATP binding was not limiting ATP hydrolysis^15^. Concanamycin A (ConcA) was used to distinguish V-ATPase activity from that of other ATPases. V-ATPase constituted over 60% of the total ATPase activity of the vacuolar vesicles. A sinusoidal AC waveform was generated either with an analogue signal generator or digitally and converted to an analogue signal using a 16-bit DA converter running at 44.1 kHz, and no difference in the effect was noted between the two methods. Metric Halo (Safety Harbor, FL 34695, USA) ULN-8 interface and converter and SpectraFoo software were used for digital signal processing and analysis. The voltage and the waveform were checked with an oscilloscope both at the input and the output of the home-built amplifier. The AC field was applied with platinum electrodes in a flat sample cell (with a cross section of 5.0 × 1.9 mm), under the same conditions as for the control. The electrodes were 4.0 mm apart, and the applied voltage was 15 V. Conductivity measurements showed that above 10 Hz the voltage drop at the electrodes is negligible, and below 10 Hz it is less than 2 V. Experiments were performed in the presence and absence of the AC field, and in the presence and absence of ConcA, each on newly prepared samples, under the same conditions as above. The difference (±ConcA) was used as a measure of V-ATPase activity, in the presence and absence of the AC field. The pore-forming ionophores, carbonylcyanide p-trifluoromethoxyphenylhydrazone (FCCP) and gramicidin A (GrA) were form Fluka (Sigma-Aldrich). They were added to the vesicle dispersion in the required quantity from stock solutions of 1.25 mM GrA and 1 mM FCCP, in ethanol.

### Statistical Analysis

Different series of experiments, each from single yeast cultures, were normalised by defining 0% as absorbance in the presence of ConcA (there was no dependence on the AC field in this case) and 100% was the absorbance without AC field and ConcA. The data sets of different series of experiments were analysed independently by fitting a single Gaussian or Lorentzian function (on a linear-log scale), with or without a linear background, depending on the width of the frequency range selected. The mean and the standard deviation of the corresponding fitting parameters are reported in the Results and Discussion section.

## Additional Information

**How to cite this article:** Ferencz, C.-M. *et al*. Oscillating Electric Field Measures the Rotation Rate in a Native Rotary Enzyme. *Sci. Rep.* **7**, 45309; doi: 10.1038/srep45309 (2017).

**Publisher's note:** Springer Nature remains neutral with regard to jurisdictional claims in published maps and institutional affiliations.

## Supplementary Material

Supplementary Video 1

Supplementary Video 2

Supplementary Video 3

Supplementary Video 4

Supplementary Video 5

Supplementary Videos

## Figures and Tables

**Figure 1:**
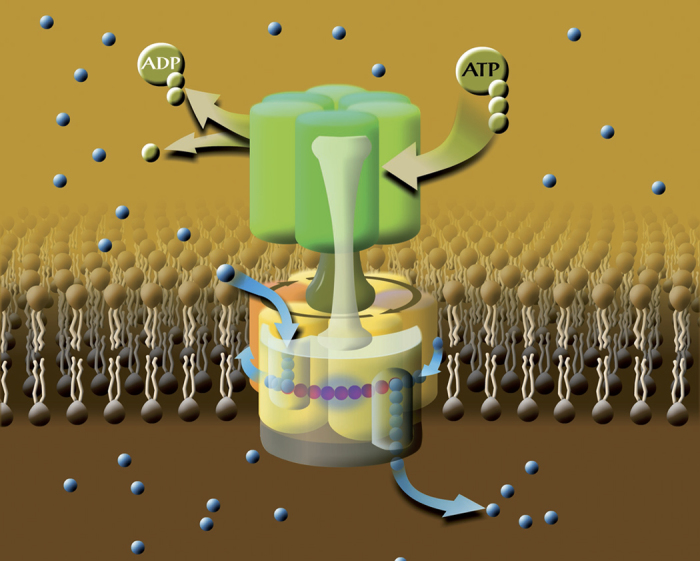
Rotary catalysis and proton pumping by V-ATPase. ATP binding and hydrolysis drives the rotor and rotation drives proton transport. Each subunit *c* (the composition of the so-called *c*-ring is actually c4c′c″) carries a proton, when in contact with lipids. Protons enter and leave the rotor in hydrophilic input and output hemichannels between the *c*-ring and subunit *a*, respectively.
