# Supplementary material for: Oscillating Electric Field Measures the Rotation Rate in a Native Rotary Enzyme
Source: Sci Rep. 2017 Mar 27;7:45309. doi: 10.1038/srep45309 (PMC5366918; doi:10.1038/srep45309)
Supplement: Supplementary Videos [file srep45309-s6.pdf]

Oscillating Electric Field Measures the Rotation Rate in a Native Rotary Enzyme

Csilla-Maria Ferencz, Pál Petrovszki, András Dér, Krisztina Sebők-Nagy, Zoltán Kóta and Tibor Páli*

Captions for Supplementary Videos

Supplementary Video 1. The rotary mechanism of V-ATPase. A mechanistic view of rotation and proton transport in V-ATPase (side view of the stator, rotor and the hemichannels) under excess substrate (ATP) conditions. Rotation takes place at the intrinsic rate of the rotary enzyme. The animation also shows the effect of a specific inhibitor that blocks rotation.

Supplementary Video 2. The rotary mechanism of V-ATPase at low substrate concentration. A mechanistic view of rotation and proton transport in V-ATPase under low substrate conditions. The average rate of rotation is limited by the on-rate of ATP binding. Not all subunits are shown.

Supplementary Video 3. Rotation and proton transport in V-ATPase in a transmembrane electric field oscillating with matching frequency. A mechanistic view of the rotation and proton transport in V-ATPase under excess substrate, but in the presence of an oscillating transmembrane electric (AC) field whose frequency matches the intrinsic rate of the 60º steps (proton cycles). If the "active" and "dead" protonation phases of the rotor have the same temporal duration, then an AC field with matching frequency and phase does not slow down the average rotation, and hence does not inhibit the enzyme. The proton-conducting intramembranous region of V-ATPase is shown from the side.

Supplementary Video 4. Rotation and proton transport in V-ATPase in a transmembrane electric field oscillating with low frequency. A mechanistic view of rotation and proton transport in V-ATPase under excess substrate, but in the presence of an AC field whose frequency is half the intrinsic rate of the 60º steps (proton cycles). Because the "wrong" phase of the AC field is longer than the "dead" protonation phase, the AC field reduces the average rate of rotation, and hence inhibits the enzyme. The proton-conducting intramembranous region of V-ATPase is shown from the side.

Supplementary Video 5. Rotation and proton transport in V-ATPase in a transmembrane electric field oscillating with high frequency. A mechanistic view of rotation and proton transport in V-ATPase under excess substrate, but in the presence of an AC field whose frequency is double the intrinsic rate of the 60º steps (proton cycles). The effect of the AC field on protonation-deprotonation of the Glu residues diminishes as its frequency exceeds the relaxation rate of the protons. The proton-conducting intramembranous region of V-ATPase is shown from the side.

— + —-
